# Sexual dysfunction is more common among men who have high sperm DNA fragmentation or teratozoopermia

**DOI:** 10.1038/s41598-022-27006-z

**Published:** 2022-12-27

**Authors:** Xiaowei Yu, XiaoYuan Zhang, Qun Wang

**Affiliations:** Department of Reproductive Medicine, Department of Prenatal Diagnosis, The First Hospital of Jilin University, Changchun, Jilin China

**Keywords:** Risk factors, Quality of life, Reproductive disorders

## Abstract

Men in couples that have experienced pregnancy loss have a higher risk of sexual dysfunction. Semen quality impairment is common in men of couples with pregnancy loss. The objective of this article is to evaluate the differences in the incidence of male sexual dysfunction in a cohort of pregnancy loss couples with different types of semen quality impairment. A cross-sectional analysis of 426 men who attended our outpatient clinic for couples’ pregnancy loss, those without genetic abnormalities were included in the final analysis covering June 2021 to October 2021. The patients were divided into 5 groups according to type of semen quality impairment: normozoospermia group (group normal; N = 161), high sperm DNA fragmentation group (group high-SDF; N = 87), isolated asthenozoospermia group (group iAstheno; N = 45), isolated teratozoopermia group (group iTerato; N = 44), and ≥ 2 abnormal sperm parameters group (group multiple; N = 89). All subjects underwent a complete physical inspection, including palpation of the male genitalia and semen analysis. Validated assessment tools for erectile dysfunction (the International Index of Erectile Function -IIEF-5) and anxiety (the seven-item Generalized Anxiety Disorder Scale- GAD-7) were also used. Men with high sperm DNA fragmentation and isolated teratozoopermia were associated with increased erectile dysfunction risk when compared with normozoospermic men, with an OR of 2.75 [1.49–5.09; *p* = 0.001] and 2.44 [1.22–5.31; *p* = 0.024], respectively. It is interesting to note that there was no difference in prevalence of erectile dysfunction between Group iAstheno and Group normal (20.0% vs. 18.0%; OR = 1.24 [0.52–2.97]; *P* = 0.625). More than half (50.6%) of the participants in Group high-SDF reported sexual intercourse less than once per week, much more than those in the normozoospermia group (23.2%, *p* < 0.05), followed by Group iTerato (44.4%) and Group multiple (46.1%). GAD-7 scores increased slightly but significantly among groups when compared with Group normal. Not surprisingly, GAD-7 scores remained higher in Group high-SDF. In males of pregnancy loss couples, men with high sperm DNA fragmentation and teratozoopermia suffer from a higher incidence of erectile dysfunction. This phenomenon is not significant in men with isolated asthenozoospermia. Proper counseling and treatment of impaired semen quality are warranted.

## Introduction

Pregnancy loss (PL) is a common complication of pregnancy and occurs in up to approximately 30% of conceptions. It refers to miscarriages that occur before 20 weeks of pregnancy^1^. Its etiology is not fully understood, more than half of couples with PL have no major risk factor^2^. For couples that have experienced PL, an extensive diagnostic workup that does not identify any etiology is difficult to accept. Both at the physical and psychological levels, PL is a huge health problem for all couples trying for pregnancy^3^.

Although PL appears to have a greater impact on women, its effects on men cannot be ignored, self-esteem and self-concept are also negatively affected the male partners with couples’ PL^3,4^. After PL, the prevalence of erectile dysfunction (ED) detected increases significantly, and is found in 19.07–27.0% of males with couples’ PL; Anxiety and depression are also significantly greater among men with couples’ PL than in the general population^5,6^. Infertility is defined as the inability to conceive following 1 year of regular unprotected intercourse. Recently, sexual dysfunction in males with infertility issues has been increasingly investigated, especially in men with impaired sperm quality. It seems clear that impaired sperm quality leads to an increasing incidence of erectile dysfunction in infertile males, and the impaired sperm quality can further aggravate the patient’s psychological state, directly affecting the patient’s sexual function^7,8^. Similarly, the presence of abnormalities and decreased spermatozoa quality is widespread in males with couple PL^9,10^. It is not known whether this relationship also exists in a population of males with couple PL.

Sperm DNA fragmentation (SDF) has now been confirmed to play an important role in adverse pregnancy outcomes^11^. However, there is no direct evidence that common sperm parameters (such as sperm count, morphology, and motility) are directly associated with PL^12^. Male partners in couples that have experienced PL frequently blame themselves for the spermatozoa disorders and attribute it to the cause of their unfavorable pregnancy outcomes. Therefore, the aim of this study is to examine whether semen quality impairment has a potential association with sexual function in a cohort of males of PL couples.

## Methods

### Ethics statement

Ethical clearance was obtained from the regional ethical committee of the First Hospital of Jilin University (21K064-001);Informed consent was obtained from all participants in compliance with the Helsinki declaration and its amendments. This study is part of a registered clinical trial on ClinicalTrials.gov (NCT04941690).

### Human subject study

**Figure 1:**
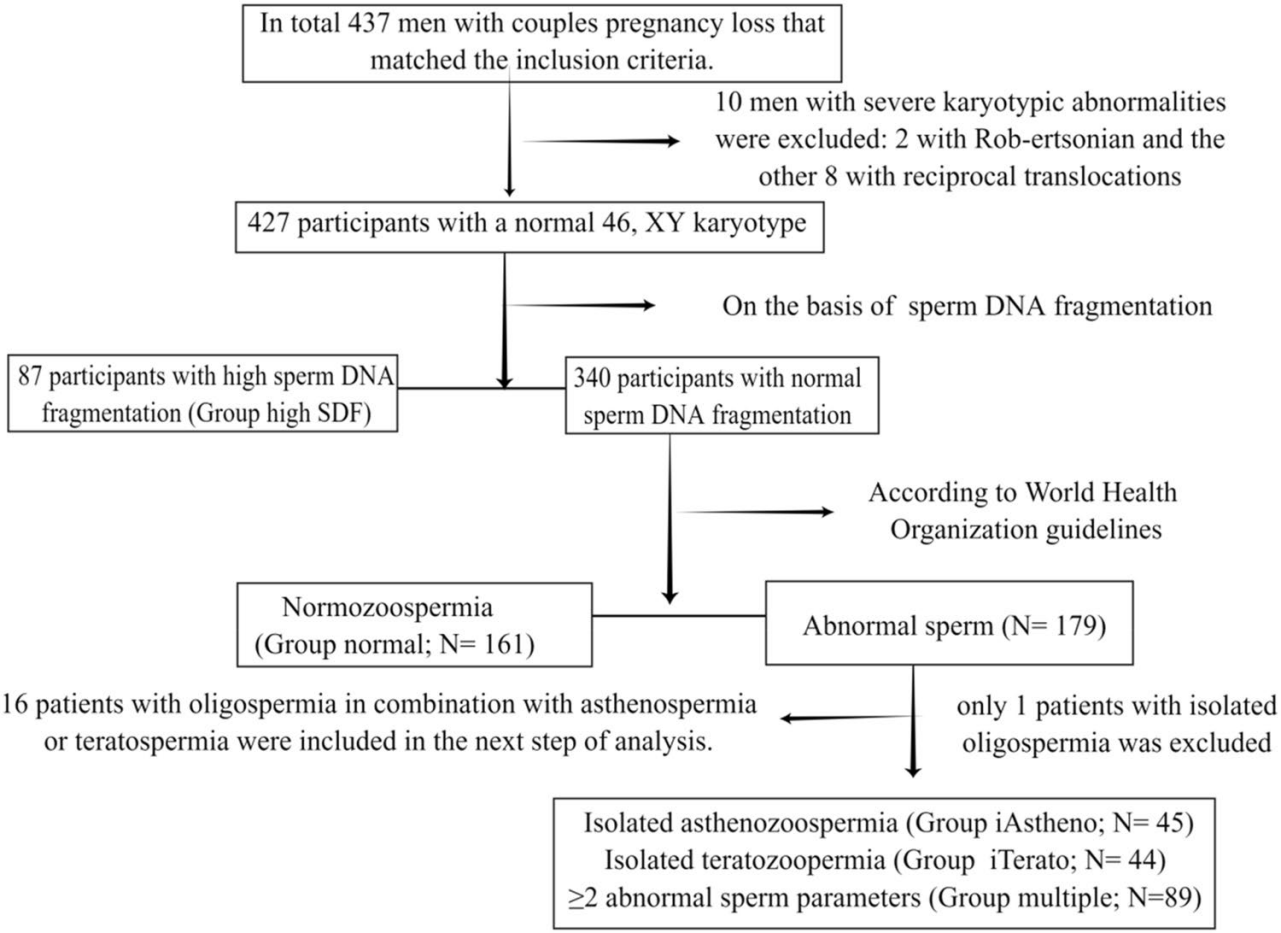
Flow chart of patient inclusion for this study.

The inclusion criteria were: (1) 20 years or more of age and seeking medical care for men in couples who have had PL; (2) men living together with their wives who had regular intercourse during the study period; (3) Couples trying to conceive for no more than 1 year. The exclusion criteria were: (1) men with a previously diagnosed with severe cardiovascular diseases, hypogonadism, or brain stroke; (2) men separated from their wives; (3) men with psychopathological conditions or who were receiving medications that may affect sexual function (such as phosphodiesterase 5 inhibitors, testosterone, and selective serotonin reuptake inhibitors); (4) men with genetic abnormalities. Based on the inclusion and exclusion criteria, we conducted a cross-sectional study evaluating a cohort of 426 male partners with couples PL from a single Reproductive Center at the First Hospital of Jilin University between June 2021 and October 2021. Patients were assigned to groups as shown in Fig. 1.

### Self-reported questionnaire

Participants completed a comprehensive demographic questionnaire on the internet, which included the five-item version of the International Index of Erectile Function (IIEF-5) for diagnosis of ED and the seven-item Generalized Anxiety Disorder (GAD-7) Scale, used to assess anxiety.

### Semen analysis and physical examination

Semen samples were collected by masturbation after three to seven days of abstinence from sexual activity. WHO guidelines were used to determine sperm quality^13^. The SDF was detected by TUNEL assay. Testicular parenchyma or varicocele were palpated during physical examinations by a solo physician.

### Data analysis

A mean ± standard deviation (SD) was calculated for normally distributed data, a median (quartile) for non-normally distributed data, and a percentage for categorical data. ANOVA or Kruskal–Wallis tests were used for comparisons of more than two groups for continuous parameters, and Categorical variables were compared using the Pearson χ^2^ test. An association between categorical parameters was calculated using the relative risk and 95% confidence interval, and a comparison was made using chi-squared test, only the variables that remained significant at a 0.05 level in the univariate analysis were included in the multivariable model. The number of pregnancy loss were finally included in the final multivariate models as adjustment factors. *P*-values < 0.05 were considered statistically significant. Data entry and analysis were performed using the IBM SPSS Statistics for Windows Version 22.0 (Armonk, NY: IBM Corp) software package.

### Ethics approval and consent to participate

The study was approved by the First Hospital of Jilin University ethics committee (21K064-001) and all participants signed informed consent.

## Results

### Participants’ characteristics

A total of 427 male partners in couples with PL met the inclusion criteria for the study. Table 1 summarizes patient characteristics.: patients were first divided into groups, depending on whether they experienced a decline in semen quality possibly correlated with couple pregnancy loss, namely the presence or absence of abnormal sperm DNA fragmentation, regardless of the presence of asthenozoospermia, teratozoopermia oroligospermia: the high sperm DNA fragmentation group (Group high-SDF; N = 87); In the next step, patients who experienced decline in semen quality unrelated to couple pregnancy loss, namely men with normal sperm DNA fragmentation, were further divided into isolated asthenozoospermia group (Group iAstheno; N = 45), isolated teratozoopermia (Group iTerato; N = 44) and ≥ 2 abnormal sperm parameters (Group multiple; N = 89) according to the semen quality. Sperm parameters without any abnormalities: the normozoospermia group (Group normal; N = 161). Statistical analysis was not performed due to the limited number of oligospermic patients in this study (only one man with isolated oligospermia was excluded). A total of 426 participants were included in the final analytic cohort. When compared with normozoospermic men, no group differences were found in the main demographic variables but were found in the number of occurrences of pregnancy losses (See Table 1). Using a 30% DNA fragmentation index threshold for high SDF^14^.

**Table 1:** Patient demographic characteristics and clinical information collected.

Demographics and clinical parameters	Group normal N = 161	Group high SDF N = 87	Group iAstheno N = 45	Group iTerato N = 44	Group multiple N = 89	*P* value
Age (years)	32.35 ± 3.93	33.62 ± 4.45	32.56 ± 5.32	32.18 ± 4.44	33.19 ± 4.08	0.150
Body-mass index (kg/m2)	25.59 ± 3.48	26.44 ± 3.03	25.22 ± 3.51	24.89 ± 3.56	25.40 ± 3.49	0.599
Current smokers (%)	29.8	29.9	31.1	18.2	40.4	0.123
Current alcohol consumption (≥ 4 drinks/week) (%)	16.1	14.9	33.3	18.2	20.2	0.093
**Education level (%)**						0.152
No higher than high school	19.9	10.3	28.9	11.4	18.0	
High school	23.0	32.2	22.2	27.3	19.1	
University and above	57.1	57.5	48.9	61.4	62.9	
**Monthly household income**						0.497
~ 5000	57.1	46.0	40.0	45.5	50.6	
5000–7000	20.5	31.0	31.1	31.8	25.8	
7000 ~	22.4	23.0	28.9	22.7	23.6	
**Night shifts (times/week) (%)**						0.409
1–2	13.0	13.8	6.7	18.2	12.4	
3–4	4.3	1.1	2.2	0	3.4	
> 4	0.6	3.4	2.2	0	0	
Mean testis volume (Prader) (ml)	13.74 ± 2.54	13.54 ± 3.19	13.41 ± 3.45	13.67 ± 2.86	12.68 ± 2.92	0.058
Clinical varicocele (Palpation) (%)	6.2	14.9	11.1	6.8	12.4	0.192
Symptoms of Prostatitis (%)	9.3	10.3	2.2	15.9	11.2	0.284
**Number of pregnancy losss (%)**						**0.002**
1	31.1	43.7	66.7	45.5	33.7	
2	49.1	40.2	15.6	40.9	48.3	
≥ 3	19.9	16.1	17.8	13.6	18.0	

Significant results are shown in bold (*p* < 0.05, paired t test).Group normal: males with normozoospermia;Group high SDF:males with high sperm DNA fragmentation. Group iAstheno: males with isolated asthenozoospermia; Group iTerato: males with isolated teratozoospermia; Group multiple: males with multiple abnormalities of sperm.

### Erectile dysfunction

**Figure 2:**
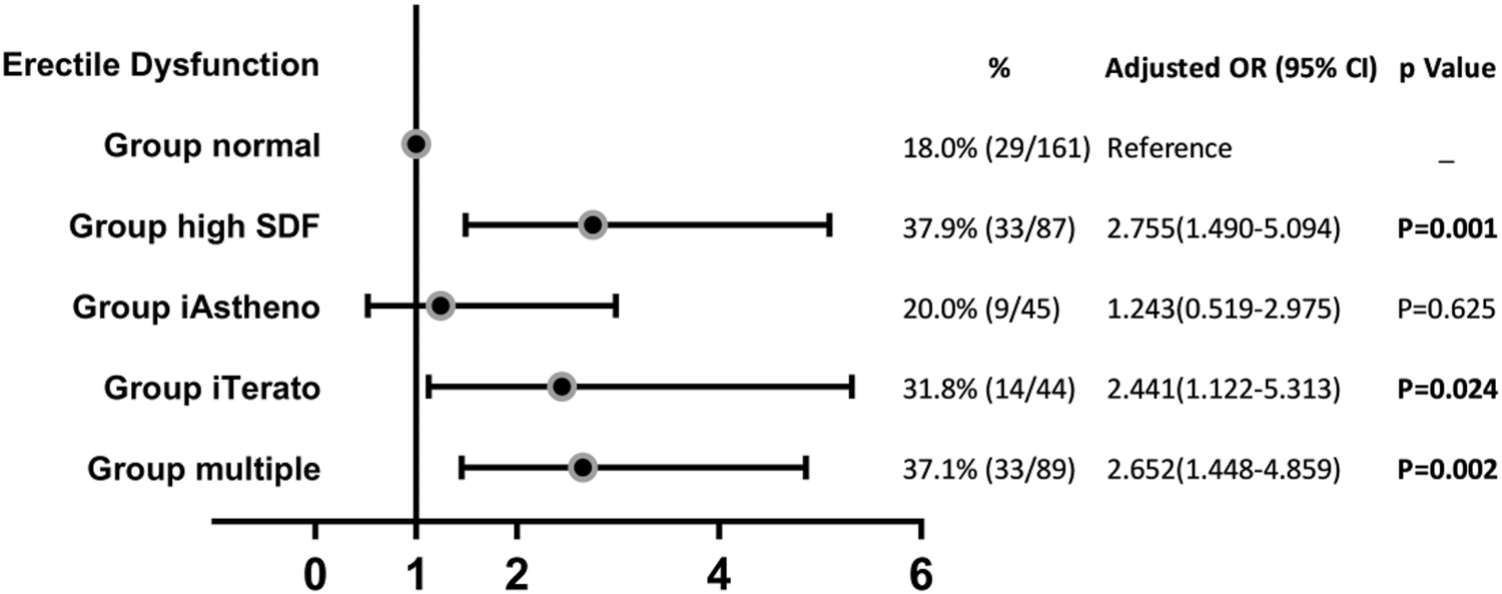
Erectile dysfunction incidence of each group are represented.

IIEF-5 scores were available from all our participants. Figure 2 shows that 37.9% of males in Group high-SDF reported ED (with an IIEF-5 score ≤ 21), followed by Group iTerato (31.8%) and Group multiple(37.1%).It is interesting to note that there was no significant difference in the prevalence of ED between Group iAstheno and Group normal (20.0% vs. 18.0%; OR = 1.24 [0.52–2.97], *P* = 0.625). Men with high SDF and isolated teratozoospermia were associated with increased ED risk when compared with normozoospermic men, with ORs of 2.75 [1.49–5.09; *p* = 0.001] and 2.44 [1.22–5.31; *p* = 0.024], respectively.

### Frequency of sexual intercourse

**Figure 3:**
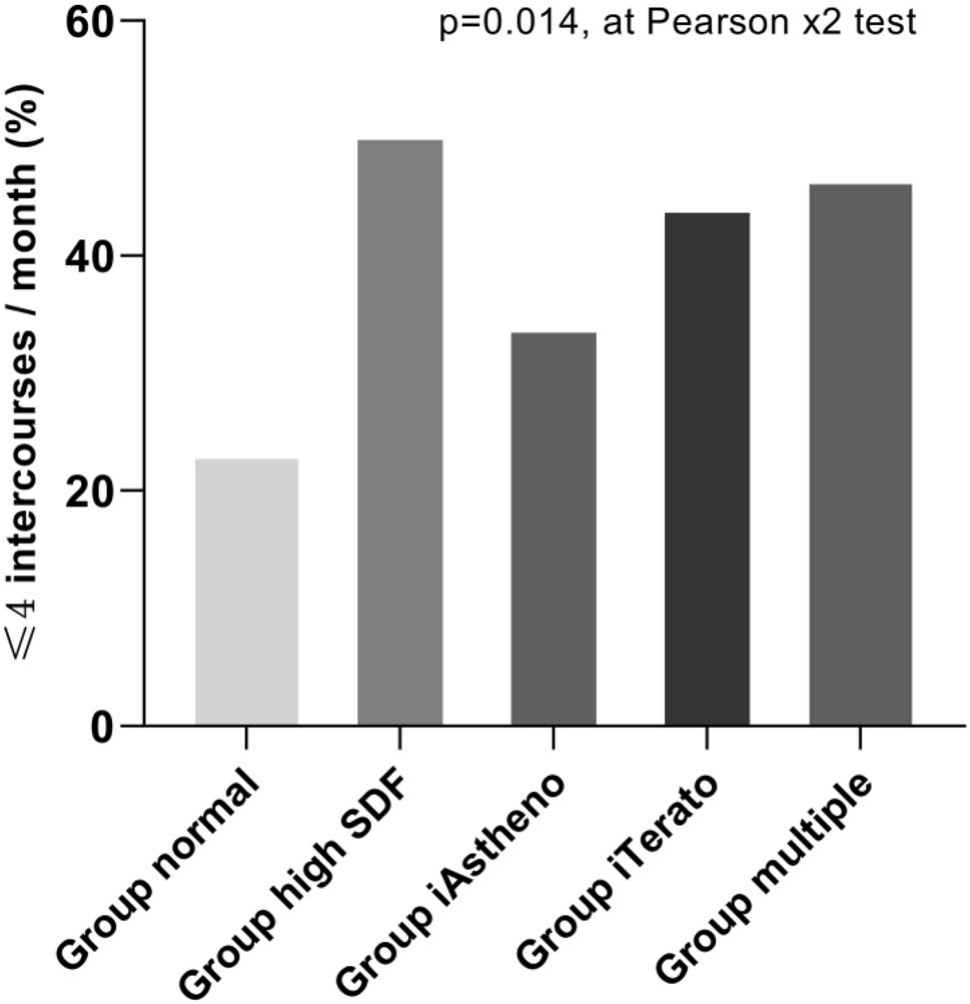
Bar graphs showing the percentages of ≤ 4 intercourses/month in different groups.

Figure 3 shows that along with semen quality impairment, there is an increased number of males’ who have sexual intercourse less than 1 time per week. More than half (50.6%) of the participants in Group high-SDF reported sexual intercourse less than once per week, much more frequent than those in the normozoospermia group (23.2%, *p* < 0.05), followed by Group iTerato (44.4%) and Group multiple(46.1%). The percentages of participants who reported sexual intercourse less than once per week were comparable between Group iAstheno (34.2%) and Group normal (22.7%).

### GAD-7 scores

**Figure 4:**
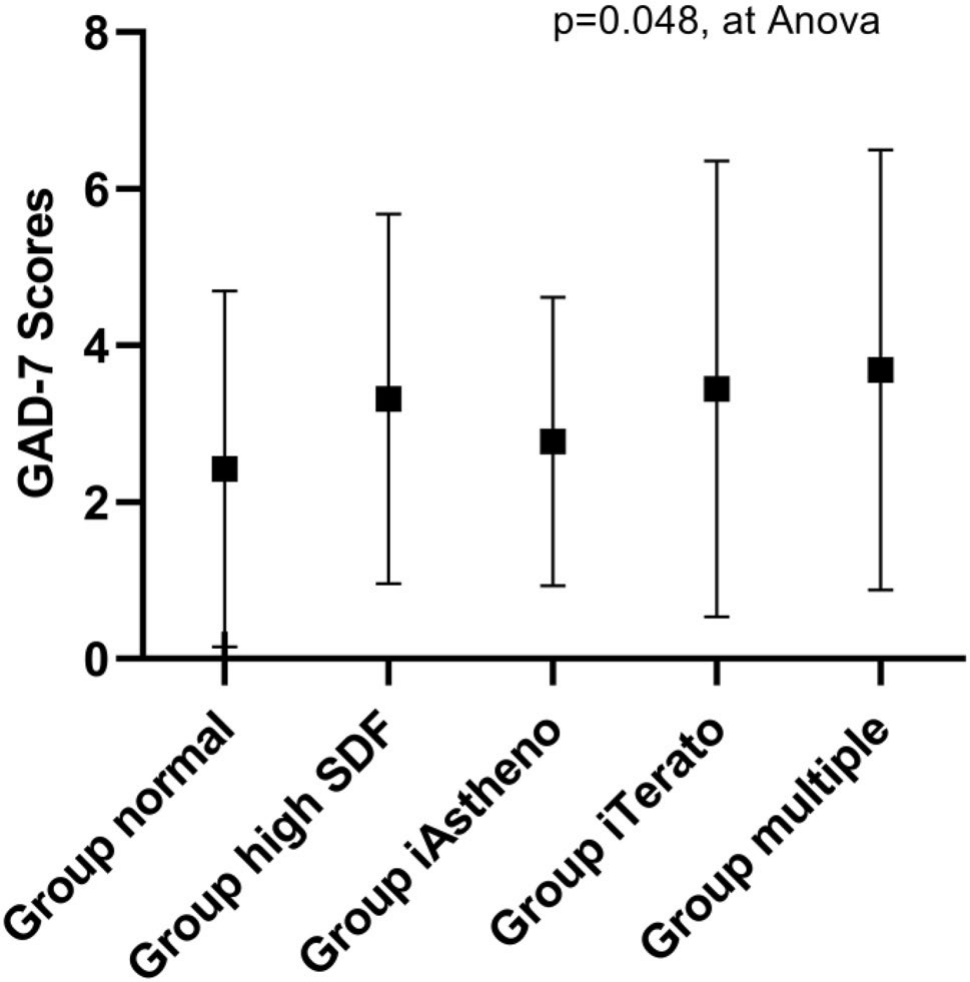
Graphs of the difference in GAD-7 scores according to groups.

Figure 4 shows that the GAD-7 scores were slightly but significantly higher among all groups when compared with Group normal. Not surprisingly, GAD-7 scores remained higher in the Group high-SDF, followed by Group iTerato and Group multiple. The GAD-7 scores were comparable between Group iAstheno and Group normal.

## Discussion

To our knowledge this is the first observational study to focus on the association between impaired sperm quality and sexual dysfunction in males of PL couples. Results were compared to normozoospermia males in the same population of similar age. Here, we have shown that impaired sperm quality negatively affects male sexual function. In particular, the prevalence of ED increases not only in males with high SDF, but also in males with isolated teratozoospermia and astheno-teratozoospermia. Interestingly, the present data do not suggest the same phenomenon in males with isolated asthenozoospermia.

As expected, we found a higher prevalence of ED in males with high SDF compared to the normozoospermic control group. Clinically, it is recommended for women to conduct detailed examinations first, followed by men screening only for karyotype abnormalities, for couples with PL. The role of sperm in PL has recently gained greater recognition, since half the genetic material in the embryo is provided by male sperm. Studies have consistently found that sperm DNA damage results in adverse pregnancy outcomes, and PL has been linked to higher SDF levels^15,16^. Those males with high SDF appear to be more willing to blame adverse pregnancy outcomes on themselves. It is also worth noting that a slight but significant increase in GAD-7 score was observed in males with high SDF in the present study. This may be related to the fact that repeated semen examination and adjuvant therapy further promotes the aggregation of psychological imbalances^17^.

Sperm quality plays a crucial role in the measures of fertility in male infertility. The evaluation of sperm quality in men with couple PL is not necessary for the etiological screening of couple PL. Although sperm DNA fragmentation may be involved in couple PL, there are no clear guidelines for recommending sperm DNA fragmentation screen. In general, there is little correlation between basic semen parameters (such as sperm count, morphology, and motility) and risk of pregnancy loss^18,19^. However, one third of pregnancies end with a miscarriage and the cause of recurring PL is unknown in about half of the cases, these couples remaining without a universal treatment recommendation^2^. For many unexplained unexplained recurrent PL, the inferior scores in the general semen parameters were very difficult to accept. In clinical work, nearly all men with teratozoopermia worried about “teratozoopermia” involved in sperm-egg binding and fertilization. In PL couples, it is easy to link the teratozoospermia with their adverse pregnancy outcomes, some saw the the teratozoospermia as heralding the beginning of the pregnancy loss. Although PL couples are repeatedly told that in the absence of treatment, up to 60–70% of couples with unexplained RPL will have success with their next pregnancy^20^. These concerns are rarely based on scientific evidence, but lacking identifiable causes in either partner is very troubling to patients, as PL is a painful experience. Great efforts have been made to improve the teratozoospermia and astheno-teratozoospermia in these patients, who are looking forward to a normal spermatozoa in preparation for pregnancy.

Sexual frequency was found to decrease markedly with the presence of sexual dysfunction based on our cohort. Ejaculation frequency is an important factor that influence semen parameters; It is widely admitted that prolonged sexual abstinence may be beneficial in semen volume and sperm concentration; It is noteworthy that the lack of ejaculation also displays adverse consequences on sperm motility and viability and SDF^21^. Spermatozoa are frequently exposed to reactive oxygen species and reactive nitrogen species during maturation and storage in epididymis before delivery to the seminal vesicle^22^. Prolonged sexual abstinence may lead to the accumulation of reactive oxygen species and further aggravated oxidative stress injury of spermatozoa^21,23^. It might be worth increasing ejaculations in order to improve sperm parameters, SDF, and consequently pregnancy outcome^24–26^.Regular intercourse at least twice a week was recommended for couples trying to conceive^27^. It was more common for male partners with ED to engage in “timed intercourse”^28^, sexual activity during the ovulation period to increase conception chances. This method may partially reduce the stress of sex life in PL couples. It may not ultimately be beneficial for accelerating pregnancy, but may increase the risk of sexual dysfunction^29^. Deliberately reducing ejaculations can further aggravate spermatozoa injury.

There are some limitations to the present study. First, lack of clinical data is an important limitation of this study. We do not have records of variables such as sex hormone profiles, which is essential not only for sperm maturation but also to male sexual function^30,31^. We may be neglecting an important bidirectional association between sex hormones and sperm quality/ sexual function. Second, in conservative cultures like China, we did not have access to female sexual function data, though male sexual dysfunction could also be aggravated by the coexistence of sexual dysfunction in the female partner^32^. In addition, the analysis was cross-sectional and hospital-based, which raises the possibility of selection bias. These findings must be interpreted with caution, and more evidence is required to validate these results.

In summary, after excluding all of the data for those subjects with genetic abnormalities from the statistical analyses, males with high SDF suffer from a higher incidence of ED. Similar associations were also observed in specific types of abnormal general semen parameters (such as teratozoospermia) but not in isolated asthenozoospermia. For males with couple PL, the subconscious idea is that teratozoopermia probably causes the fetal abnormalities. Increase in GAD-7 scores and lack of sex life was observed in these patients, men with semen quality impairment tend to attribute sexual dysfunction to psychological factors. Thus, proper counseling and treatment of impaired semen quality not only implies a direct clinical benefit for the males with couple PL, but may even help optimize pregnancy outcomes.

## Data Availability

The datasets used and/or analyzed in this study are available from the corresponding authors upon reasonable request.

## Abbreviations

PL: Pregnancy loss
SD: Sexual dysfunction
TI: Timed intercourse
ED: Erectile dysfunction
IIEF-5: International index of erectile function
GAD-7: 7-Item generalized anxiety disorder scale
SDF: Sperm DNA fragmentation
ANCOVA: Analysis of covariance
SD: Standard deviation
RPL: Recurrent pregnancy loss
